# Anhedonia as a Potential Risk Factor of Alzheimer’s Disease in a Community-Dwelling Elderly Sample: Results from the ZARADEMP Project

**DOI:** 10.3390/ijerph18041370

**Published:** 2021-02-03

**Authors:** David Vaquero-Puyuelo, Concepción De-la-Cámara, Beatriz Olaya, Patricia Gracia-García, Antonio Lobo, Raúl López-Antón, Javier Santabárbara

**Affiliations:** 1 Psychiatry Service, Hospital Clínico Universitario Lozano Blesa, 50009 Zaragoza, Spain; 648724@unizar.es (D.V.-P.); edelacam@unizar.es (C.D.-l.-C.); 2 Department of Medicine, Psychiatry and Dermatology, Universidad de Zaragoza, 50009 Zaragoza, Spain; alobo@unizar.es; 3 Instituto de Investigación Sanitaria de Aragón (IIS Aragón), 50009 Zaragoza, Spain; rlanton@unizar.es (R.L.-A.); jsantabarbara@unizar.es (J.S.); 4 Centro de Investigación Biomédica en Red de Salud Mental (CIBERSAM), Ministry of Science and Innovation, 28029 Madrid, Spain; beatriz.olaya@pssjd.org; 5 Research, Innovation and Teaching Unit, Parc Sanitari Sant Joan de Déu, Universitat de Barcelona, 08007 Barcelona, Spain; 6 Psychiatry Service, Hospital Universitario Miguel Servet, 50009 Zaragoza, Spain; 7 Department of Psychology and Sociology, Universidad de Zaragoza, 50009 Zaragoza, Spain; 8 Department of Microbiology, Pediatrics, Radiology and Public Health, Universidad de Zaragoza, 50009 Zaragoza, Spain

**Keywords:** anhedonia, Alzheimer’s disease, psychopathology, risk factor, neuropsychiatry, community study

## Abstract

(1) Introduction: Dementia is a major public health problem, and Alzheimer’s disease (AD) is the most frequent subtype. Clarifying the potential risk factors is necessary in order to improve dementia-prevention strategies and quality of life. Here, our purpose was to investigate the role of the absence of hedonic tone; anhedonia, understood as the reduction on previous enjoyable daily activities, which occasionally is underdetected and underdiagnosed; and the risk of developing AD in a cognitively unimpaired and non-depressed population sample. (2) Method: We used data from the Zaragoza Dementia and Depression (ZARADEMP) project, a longitudinal epidemiological study on dementia and depression. After excluding subjects with dementia, a sample of 2830 dwellers aged ≥65 years was followed for 4.5 years. The geriatric mental state examination was used to identify cases of anhedonia. AD was diagnosed by a panel of research psychiatrists according to Diagnostic and Statistical Manual of Mental Disorders, Fourth Edition (DSM-IV) criteria. A multivariate survival analysis and Cox proportional hazards regression model were performed, and the analysis was controlled by an analysis for the presence of clinically significant depression. (3) Results: We found a significant association between anhedonia cases and AD risk in the univariate analysis (hazard ratio (HR): 2.37; 95% CI: 1.04–5.40). This association persisted more strongly in the fully adjusted model. (4) Conclusions: Identifying cognitively intact individuals with anhedonia is a priority to implement preventive strategies that could delay the progression of cognitive and functional impairment in subjects at risk of AD.

## 1. Introduction

Dementia constitutes a serious public health challenge worldwide. By 2030, 82 million people are expected to develop dementia, and that number is expected to reach over 152 million in 2050 [1,2]. Cognitive impairment is a core symptom, along with the presence of functional impairment in the context of a gradual and progressive clinical evolution [3]. In this context, Alzheimer’s disease is the most frequent subtype of dementia, and it has a huge impact not only on the patient (institutionalization, risk of mortality, etc.) and the caregivers (such as psychiatric complications) [4,5], but also on the socioeconomic dimension [1,2]. The lack of an effective treatment highlights the need for the identification of potential psychopathological risk factors, and the improvement of prevention strategies to decrease its incidence and improve quality of life. Nevertheless, longer periods of observation are needed, with a sufficient frame of time to reduce the risk of AD, so further investigations are fundamental.

The association between clinically significant depression and dementia is well known [6,7]. This is specifically true for severe and major episodes [8], with severe cases of depression being four times at greater risk of Alzheimer’s disease (AD) compared with subjects without depression [9]. There are two main clinical manifestations to diagnose a major depressive disorder: hypothymia and/or anhedonia [3]. Hypothymia is understood as a decrease in the baseline holothymic state, which includes the affective tone and emotional reactivity. Anhedonia is conceptualized as the presence of a reduction or lack of interest or enjoyment in previously pleasurable projects, which could be understood as a type of negative non-cognitive symptom [10] or a mood-related one. 

Previous studies [11,12,13,14,15,16] suggest that anhedonia could be either a potential preclinical sign of cognitive decline or a psychopathological risk factor of dementia. However, the potential association of depression-spectrum symptoms and Alzheimer’s disease is still unknown. Furthermore, the heterogeneity of the definition of anhedonia across different studies has led to mixed results.

In the Zaragoza Dementia and Depression (ZARADEMP) project, a longitudinal epidemiological study of dementia and depression conducted in community-dwelling older adults, the authors found that anhedonia was frequently observed among cases of Alzheimer’s disease (46.8% vs. 2.5% in non-demented participants) [10,11]. Other epidemiological studies suggest that the presence of anhedonia implies a two-times greater risk of mild cognitive impairment and, remarkably, this symptom increases the probability of dementia by a factor of five [15], particularly Alzheimer’s disease [16]. These results may be supported by studies showing a link between apathy and amyloid burden in mild cognitive impairment [12], and by those reporting an association of “apathy-anhedonia” symptoms and hypometabolism in certain brain regions and hippocampal volume reductions [13], with a probable proinflammatory role of the microglia [14]. 

Although a link between anhedonia and the risk of dementia is strongly suggested [15,16], further epidemiological studies with representative samples of cognitively intact people are needed. Thus, our purpose was to investigate the relationship between anhedonia and the risk of Alzheimer’s disease in cognitively intact community-dwelling elderly people without depression.

## 2. Materials and Methods

### 2.1. Sample

We used data from the ZARADEMP project [17,18], a longitudinal population-based study conducted in Zaragoza, Spain. The main objective was to estimate the incidence and risk factors for dementia and depression in adults aged 55 years or older. Further information about objectives and design has been published previously [18].

Briefly, a random sample of community-dwelling older people was drawn from the Spanish official census list of 1991, which included institutionalized individuals and was stratified with proportional allocation by age and gender. The refusal rate was 20.5%; 4803 individuals participated at baseline (Wave I started in 1994). Individuals with all-cause dementia were excluded from the two follow-up waves (second wave in 1997 and third wave in 1999) because we were interested in cognitively intact individuals, so that stringent criteria were applied. Additionally, subjects with “subsyndromal” dementia at baseline, according to the Geriatric Mental State (GMS) Automated Geriatric Examination for Computer Assisted Taxonomy package (AGECAT) criteria [19], were also excluded. 

For ease of comparison with the existing literature, we focused on participants aged 65 years and older. Additionally, we focused on those participants who participated both at baseline and in the second wave, resulting in a final sample of 1642 participants for statistical analysis [20].

### 2.2. Procedure

In phase I, the ZARADEMP interview was conducted by well-trained and regularly supervised lay-interviewers at each participant’s residence, or in hospital if this option was preferred. 

This interview incorporated standardized and validated Spanish versions of the following international instruments: Mini-Mental State Examination (MMSE) [21], Geriatric Mental State (GMS) [19], and History and Aetiology Schedule (to asses psychiatric history) [22], and the Katz Index [23] and Lawton and Brody Scale [24] (to assess basic and instrumental activities of daily living (ADLs), respectively). The European Studies of Dementia (EURODEM) Risk Factors Questionnaire [19,25] was used to consider medical history. 

In phase II, a trained research psychiatrist reassessed participants to confirm the suspected clinical diagnosis of dementia and/or depression. The validity of this approach has been established [26]. A similar procedure was implemented in the second and third (2.5 and 4.5 years later, respectively), in which interviewers were not aware of the results of the baseline interview described beforehand. A more detailed account of the methods has been published elsewhere [17].

### 2.3. Ethics

The Ethics Committee of Research of Aragón (CEICA) approved the project in accordance with Spanish law. The Declaration of Helsinki principles [27] of written informed consent, confidentiality, and privacy were maintained throughout the project.

### 2.4. Clinical Measurements

#### 2.4.1. Alzheimer’s Disease Assessment and Diagnosis

At the end of the baseline assessment, identified “cases” of dementia and “subcases” of dementia (according to GMS-criteria) were excluded from the follow-up waves. Participants were considered “probable cases” on the basis of a GMS threshold “global” score (1/2) and/or MMSE (23/24) standard cutoff points. 

A panel of four research psychiatrists made the final diagnoses of cases of dementia. The validity of this diagnostic process has also been proven [26]. Variables in the ZARADEMP interview were operationalized to conform to the Diagnostic and Statistical Manual of Mental Disorders, Fourth Edition (DSM-IV) [3] criteria, used to diagnose cases. Agreement by at least three out of the four psychiatrists was necessary for a diagnosis of “incident” dementia and “type of dementia” (e.g., AD or vascular dementia). 

To document the accuracy of the panel of psychiatrists, detected cases were invited to a hospital diagnostic work-up, which included a neurological examination and neuroimaging. For the purposes of this paper, we focused on AD cases. Nacional Institute of Neurological and Communicative Disorders and Stroke-Alzheimer´s Disease and Related Disorders Association (NINCDS-ADRDA) criteria were applied to diagnose AD.

#### 2.4.2. Anhedonia Assessment and Diagnosis

Geriatric Mental State (GMS) is a semi-structured standardized clinical interview for assessing the mental state of elderly people [19]. It includes melancholic items, such as the symptom of anhedonia, and a computerized diagnostic program, namely AGECAT. This consists of a set of computer algorithms to analyze the GMS data, which was applied to reach the psychiatric diagnosis [19,28]. The reliability and validity of the Spanish version of the GMS-AGECAT procedure has been reported elsewhere [28].

For the purpose of this study, the symptom of anhedonia, which appears in the DSM-IV and in the International Classification of Diseases (ICD-10) as a melancholic or a negative-non-cognitive clinical manifestation, was operationalized using the information from the GMS through the following questions: Do you take pleasure in anything? What do you like doing lately? Has there been any change? We followed standard GMS procedures to use binary variables throughout the calculation process. To this end, we recorded “0” (when the symptom was absent), and scores “1” (symptom present, but mild or not frequent) and “2” (symptom frequent and/or severe) were collapsed. 

### 2.5. Covariates

The potential confounders assessed at baseline included sociodemographic characteristics (age, gender [29], educational level, marital status [30,31,32], and living alone), medical risk factors based on the medical history obtained using the EURODEM Risk Factors Questionnaire [33] (vascular disease [34], hypertension, and diabetes), cognitive state (MMSE score), functional disability (ADL [35]), body mass index (BMI), and clinically significant depression [9,36,37].

### 2.6. Statistical Analysis

Differences between baseline characteristics according to anhedonia status were assessed using two-tailed chi-square tests on categorical data, and differences in variables with approximately normal distributions were assessed using a two-tailed t-test. Standard procedures were used to calculate the incidence rate and incidence rate ratio (IRR). 

The follow-up period ended in the second follow-up examination (third wave) for the cognitively intact individuals, at the date of invitation for refusals, at the date of moving away or death (based on actual data from the Civil Registry, Padrón Municipal de Habitantes de Zaragoza), or at the time of onset of dementia for cases. The time of onset of dementia was estimated to be the time from baseline to the midpoint between diagnosis and the previous examination.

We built Kaplan–Meier survival curves according to the anhedonia status. The probability of AD-free survival across groups was assessed by means of the Tarone-Ware test. 

We used the Cox proportional hazards regression model to calculate the risk of participants who had the psychopathological symptom of anhedonia (cases), compared with those without this clinical manifestation (no cases), for experiencing AD, and with age as timescale with delayed entry [38]. To explore the mechanisms explaining the association between anhedonia and the risk of developing AD, we used a series of models in which we gradually controlled for the potential modifiers previously mentioned. We confirmed the assumption of proportional hazards by means of the Therneau and Grambsch test [39]. 

Statistical analyses were conducted using R software (http://www.r-project.org) (R Foundation for Statistical Computing, Vienna, Austria), with the epiR package to analyze epidemiologic data, and the survival and survminer packages for the survival analyses.

## 3. Results

**Figure 1:**
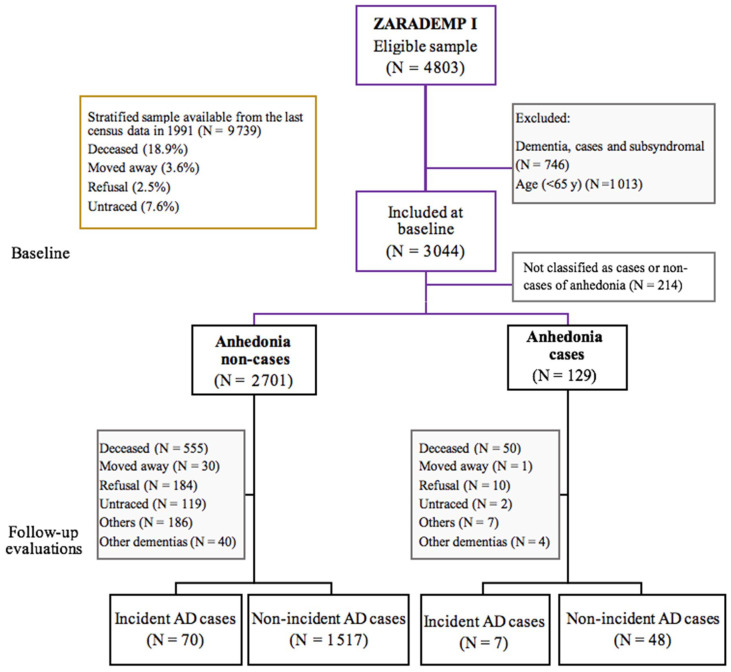
Zaragoza Dementia and Depression (ZARADEMP) project study flow chart. Notes: AD—Alzheimer’s disease.

Our final sample included 2830 older adults free of dementia at baseline (median 4.4 years; interquartile range: 2.8–4.9 years). During the follow-up period (4.5 years), 1565 (55.3%) were non-incident AD cases, 77 (2.7%) were incident AD cases, 44 (1.5%) were incident cases of other dementias, 605 (21.4%) died, and 539 (19%) were lost (by refusal to take part, changing residence, or being impossible to contact; Figure 1). Those lost or dead during follow-up were older (*p* < 0.001) and more likely to be illiterate than those re-evaluated; the MMSE scores were also lower among those lost or dead (*p* < 0.001; data not shown).

Table 1 shows the sociodemographic characteristics at baseline according to the AD incidence status. Participants with incident AD were significantly older, were more likely to be female, formerly married, or to have a lower educational level, and were more likely to have depression, to perform worse cognitively, to have anhedonia, and to have more functional disabilities than participants without AD.

**Figure 2:**
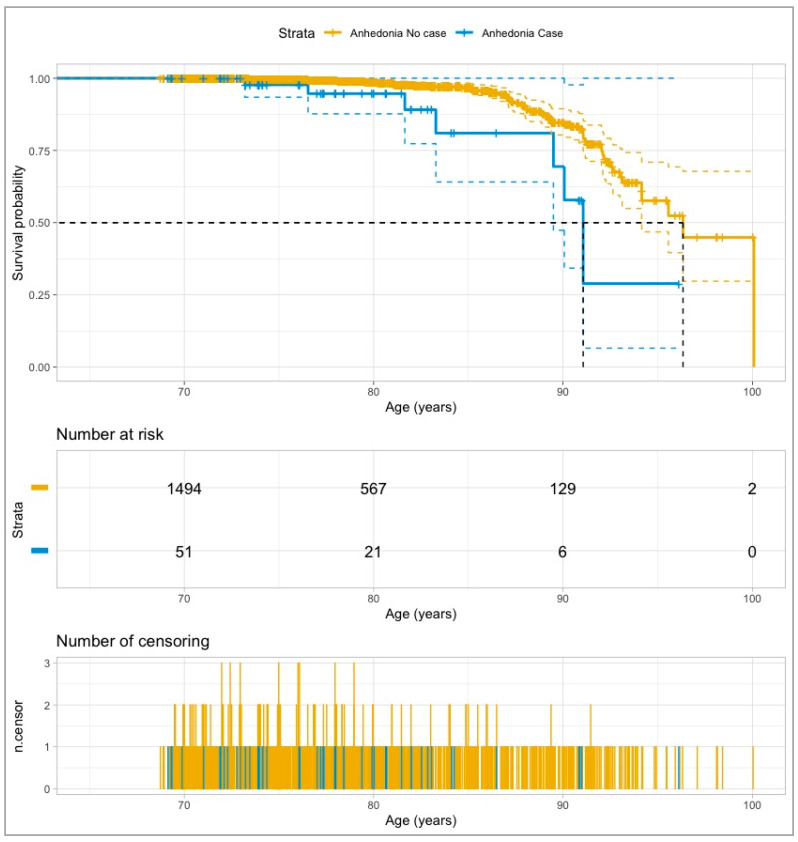
Survival curves for incident AD according to the Anhedonia status at baseline. *Notes:* Strata: Anhedonia group. Survival median (age for survival probability = 0.50 in each anhedonia group) is represented by a dashed black line. n.censor is the number of censored data (participants who died or were lost during follow-up).

The crude comparison of the survival curves according to anhedonia status (Figure 2) revealed a more favorable survival probability in the no case group (Tarone-Ware test; *p* = 0.003). In fact, the age survival median was 96.3 years (95% CI: 94.2–100) for no case participants, significantly higher than 91.1 years (95% CI: 89.5–100) for the cases of anhedonia.

Table 2 shows a significant association between “anhedonia cases” at baseline and AD risk. Compared with no cases, the proportion of incident cases and the incidence rate of AD were higher among anhedonia cases (IRR = 2.5; *p*-value = 0.021). Table 2 also shows the results of the Cox regression analysis for the risk of AD associated with anhedonia status. The risk of AD was almost 2.5-fold higher in anhedonia cases compared with no cases when the socio-demographics factors were controlled for (hazard ratio (HR): 2.37; 95% CI: 1.04–5.40). This association persisted or was slightly higher in the fully adjusted model.

## 4. Discussion

Our observations suggest that the appearance of anhedonia in cognitively intact and non-depressed older people increases the risk of developing AD by 2.5 times, compared with those without anhedonia and after controlling for the effect of sociodemographic factors. With the aim of uncovering the multicausality and complexity of Alzheimer’s disease, this is the first study to report a significant association between the presence of anhedonia (independent of a depressive disorder) and risk of AD.

Studies focusing on anhedonia as part of a mood disorder suggest that anhedonia predicts a significant worse clinical prognosis in subjects with acute coronary syndrome [40], where it doubles the risk of suffering from another episode again [41,42]. Previous literature has also suggested anhedonia to be a predictor of death in patients with systolic heart failure [43], patients undertaking a coronary stent implantation [44], and community-dwelling adults with type-2 diabetes [45].

Literature about anhedonia as a symptom (outside the context of a mood episode) has shown that subjects with anhedonia have a higher risk of cardiovascular diseases [16,40,41,42,43,44], type-2 diabetes [46], and mild cognitive impairment (MCI) [15].Moreover, some experimental works based on animal models suggest that anhedonia may increase risk of AD [47,48,49]. 

The presence of the symptom of anhedonia without a major depressive disorder was associated with a six-times higher risk of conversion to AD in individuals with MCI. Furthermore, this association has not been found in depressed people with an appropriate hedonic tone [16]. A similar study conducted in the Korean Longitudinal Study on Cognitive Aging and Dementia (KLOSCAD), a prospective multicenter cohort study in adults aged 60 years or older [50], found that non-demented and non-clinically depressed participants with anhedonia were at a five-times greater risk of global dementia than those with an appropriate hedonic tone [15]. This study, though, did not report the specific risk by subtypes of dementia (e.g., AD). 

Our study is the first to analyze the association between anhedonia and Alzheimer’s disease in cognitively unimpaired elderly people without clinical depression. In contrast with previous studies [45] that assessed anhedonia by means of self-reported questionnaires, we used a semi-structured standardized ZARADEMP clinical interview where GMS-AGECAT was included, which is specific for a psychogeriatric population. Controlling for clinically significant depression and the use of international and reliable clinical instruments should increase the confidence in our results, especially in those concerning the independent role of anhedonia from depression [15,16] as a genuine risk factor of AD. 

Some specific neurobiological pathways might be involved in the association between anhedonia and AD. Firstly, we reported some evidence that found that anhedonia increases the risk of diabetes and cardiovascular disease; both have been consistently associated with an increased risk of AD [45]. However, we controlled our analysis for those risk factors, and the association between anhedonia and incident AD were independent of them. Otherwise, loss of pleasure is related with reward-related deficits [51], and different brain structures have been suggested to be involved, such as the prefrontal cortex (ventromedial and orbitofrontal) [52], dorsal and ventral striatum [53] (nucleus accumbens) [54,55,56], area tegmental ventral, amygdala [57], habenula [58], primarily mediated by dopaminergic and glutamatergic systems [51,59,60]. From an experimental approach, a “vicious cycle of stress” is described [47], with anhedonia being a source of emotional discomfort in the context of vulnerability to stress [61]. This clinical manifestation is capable of reducing the hippocampal volume [12,13] and disrupting microglia function through the activation of the brain neuroinflammation (a cytokine cascade and oxidative stress) [14,48], which determines an increased risk of developing AD [49]. In turn, neuroinflammation might increase the severity of anhedonic symptomatology [47]. 

It is still unknown if anhedonia is a genuine risk factor of AD or rather an incipient prodromal symptom of AD. As we were primarily interested in the implications of anhedonia in a cognitively intact sample, we excluded those cases with dementia or mild cognitive deficits at baseline. Additionally, in order to study the role of anhedonia outside the context of a major depressive episode, we controlled our analysis for clinically significant depression at baseline, as well as a history of depression and/or treatment with antidepressants. Thus, our findings seem to suggest that anhedonia as a symptom might be a potential independent risk factor of AD.

Nevertheless, anhedonia could be a symptom of subsyndromal or minor depression, which consists of depressive symptoms that do not meet the criteria for clinically significant or major depression. Subsyndromal depression has been associated with poorer outcomes and an increased dementia global risk [8]. Anhedonia could also be a symptom of apathy syndrome, which has consistently been found to be a risk factor of progression to dementia in different clinical samples [62]. However, anhedonia refers specifically to the affective component of apathy, which could respond better to treatment with antidepressants than behavioral apathy. Future studies could attempt to uncover to a better understanding of the context of anhedonia as a risk factor of AD. Furthermore, they should also determine if the detection of anhedonia in non-depressed subjects and its treatment could have a preventive effect on AD risk. 

Some of the limitations of this study are attributable to a relatively short follow-up period. Longer periods of time might be needed to clarify whether anhedonia is a genuine risk factor of dementia or rather a prodromal symptom. We did not analyze biochemical markers in the blood, serum, or cerebrospinal fluid, because our study has an epidemiological basis and our main objective was to document the association between diverse risk factors and incident cases of dementia in the elderly population, using several instruments specific for psychogeriatric dwellers [18]. Nevertheless, biochemical markers might have helped us to understand the different pathophysiological and psychopathological pathways underlying mechanisms between anhedonia and AD a bit better, so further research is needed. In addition, it is difficult to make a comparison between our results and those of other epidemiological studies, because of the diverse assessment instruments and definitions of anhedonia [55]. Anhedonia is sometimes not properly operationalized or considered to be equivalent to a reduction in positive affect or a loss of interest and hopefulness [43]. 

## 5. Conclusions

In conclusion, our study suggests that anhedonia, independent of a mood disorder, is a probable psychopathological risk factor of AD. Thus, measuring this symptom in daily clinical practice could provide a helpful, rapid, and easy tool for clinicians to identify those cases at risk of AD. Those subjects at risk should be kept under observation over time and offered effective dementia prevention strategies [2,6,37,63,64] in order to prevent progression to AD. 

## Figures and Tables

**Table 1:** Baseline characteristics according to incident AD status.

	Follow-Up AD Status		
Variables	Non-Incident AD (N = 1565)	Incident AD (N = 77)	*p*-Value
**Socio-demographic characteristics**			
Age (years)	73.4 (6.5)	84.1 (6.6)	<0.001
Female sex	874 (55.8%)	55 (71.4%)	0.010
Education (years)	7.6 (3.9)	5.7 (3.5)	<0.001
Marital status (ref. single)			<0.001
Married/in couple	1001 (63.9%)	23 (29.9%)	
Formerly married	409 (26.1%)	53 (68.8%)	
**Psychopathological risk factors**			
Depression	110 (7%)	10 (13%)	0.082
Anhedonia	48 (3%)	7 (9%)	0.011
Behavioural risk factors			
BMI	27.1 (6.3)	26.1 (5.1)	<0.001
**Vascular risk factors**			
Diabetes	192 (12.2%)	8 (10.4%)	0.770
Hypertension	1116 (71.3%)	49 (63.6%)	0.181
Previous vascular disease	110 (7%)	5 (6.5%)	0.304
**Functional and cognitive status**			
Basic ADLs	69 (4.4%)	13 (16.9%)	<0.001
Instrumental ADLs	131 (8.4%)	32 (41.6%)	<0.001
MMSE score	30.8 (2.8)	27.4 (2.9)	<0.001

Notes: AD (Alzheimer Disease); ADLs: Activities of Daily Living; BMI: Body Mass Index. Data are given as mean (standard deviation) or frequency (%).

**Table 2:** Anhedonia status at baseline and risk of AD.

					UNIVARIATE MODEL		MULTIVARIATE MODEL	
ANHEDONIA STATUS AT BASELINE	No. (%) of AD Incident Cases	Person-Years	IR (per 1000 Person-Years) (95% CI)	IRR (95% CI)	HR (95% CI)	*p*-Value	HR (95% CI)	*p*-Value
No case (n = 1587)	70 (4.4%)	10,529	6.6 (5.2–8.4)	1	1		1	
Case (n = 55)	7 (12.7%)	424	16.5 (6.6–34.0)	2.48 (1.14–5.40)	2.37 (1.04–5.40)	0.039	2.95 (1.03–8.47)	0.043

Notes: AD—Alzheimer’s disease; IR—incidence rate; IRR—incidence rate ratio; HR—hazard ratio. Reported HR of AD is related to no cases. CIs and *p*-values related to HR were from a “normal approximation” of Wald’s χ2 test with 1 df. Model 1: included anhedonia plus terms for socio-demographic characteristics (sex, years of education, and marital status). Model 2: included additional terms for vascular risk factors (body mass index, previous vascular disease, hypertension, and diabetes), depression, disability, and cognitive status at baseline (MMSE).
